# The Wnt Co-Receptor Lrp5 Is Required for Cranial Neural Crest Cell Migration in Zebrafish

**DOI:** 10.1371/journal.pone.0131768

**Published:** 2015-06-29

**Authors:** Bernd Willems, Shijie Tao, Tingsheng Yu, Ann Huysseune, Paul Eckhard Witten, Christoph Winkler

**Affiliations:** 1 Department of Biological Sciences, National University of Singapore, Singapore, Singapore; 2 Centre for Bioimaging Sciences (CBIS), National University of Singapore, Singapore, Singapore; 3 Biology Department, Ghent University, Ghent, Belgium; Texas A&M University, UNITED STATES

## Abstract

During vertebrate neurulation, cranial neural crest cells (CNCCs) undergo epithelial to mesenchymal transition (EMT), delaminate from the neural plate border, and migrate as separate streams into different cranial regions. There, they differentiate into distinct parts of the craniofacial skeleton. Canonical Wnt signaling has been shown to be essential for this process at different levels but the involved receptors remained unclear. Here we show that the frizzled co-receptor low-density-lipoprotein (LDL) receptor-related protein 5 (Lrp5) plays a crucial role in CNCC migration and morphogenesis of the cranial skeleton. Early during induction and migration of CNCCs, *lrp5* is expressed ubiquitously but later gets restricted to CNCC derivatives in the ventral head region besides different regions in the CNS. A knock-down of *lrp5* does not interfere with induction of CNCCs but leads to reduced proliferation of premigratory CNCCs. In addition, cell migration is disrupted as CNCCs are found in clusters at ectopic positions in the dorsomedial neuroepithelium after *lrp5* knock-down and transient CRISPR/Cas9 gene editing. These migratory defects consequently result in malformations of the craniofacial skeleton. To date, Lrp5 has mainly been associated with bone homeostasis in mammals. Here we show that in zebrafish, *lrp5* also controls cell migration during early morphogenetic processes and contributes to shaping the craniofacial skeleton.

## Introduction

Neural crest cells (NCCs) are multipotent precursor cells that are specified at the boundary between neural plate and epidermis upon induction by growth factors such as Wnts, BMPs, FGFs and retinoic acid (RA) [1]. Committed NCCs undergo an epithelial to mesenchymal transition (EMT) before delaminating from the neural plate and migrating ventrally along distinct routes. Depending mostly on extrinsic cues derived from targeting tissues, they differentiate into various cell types and tissues such as neurons of the enteric and peripheral nervous system, endocrine and para-endocrine derivatives and pigment cells [2]. In the amniote head, cranial neural crest cells (CNCCs) migrate ventrally from hindbrain rhombomeric regions into the pharyngeal arches and the frontonasal process where they give rise to facial cartilage, bone and connective tissue. Three characteristic major streams can be distinguished: The mandibular, hyoid and branchial stream [3]. The branchial stream originates from the neuroepithelium of rhombomeres 6 to 8 and invades the 3^rd^ to 7^th^ pharyngeal arches. In actinopterygians, each of these five arches will give rise to one of the five ceratobranchials, in addition to other splanchnocranial elements. While several factors that control arch formation have been uncovered, particularly in zebrafish [4,5], the detailed mechanisms linking CNCC proliferation and migration to differentiation remain unclear.

A number of studies revealed that canonical Wnt signaling is one of the crucial signal transduction pathways involved in all NCC related processes that take place in the course of development [6]. In *Xenopus laevis*, it was shown that activation of Wnt signaling induces ectopic neural crest [7]. In contrast, blocking Wnt signaling by misexpression of GSK3β [8], dominant-negative Wnt8 [9], truncated Tcf3 [10] or Nkd [11] resulted in the disruption of neural crest formation. Thus, Wnt signaling is important for induction of NCCs. In zebrafish, a knock-down of Wnt8 by antisense Morpholinos blocked early NCC induction and a critical phase for NCC induction has been observed by expression of truncated Tcf under control of a heatshock-inducible promoter [10]. Wnts furthermore regulate proliferation and subsequent delamination of NCCs from the dorsal neuroepithelium in chicken [12]. A role in migration has also been suggested since LiCl-mediated GSK3β inhibition prevents cell migration and blocks cell-matrix adhesion in cultured neural crest cells [13]. In *Xenopus*, a role for the frizzled co-receptor low-density-lipoprotein (LDL) receptor-related protein 6 (Lrp6) has been suggested for NCC induction since its misexpression expands the neural crest. In contrast, overexpression of a truncated dominant-negative form of Lrp6 seemed to reduce the number of neural crest cells [14].

Gene expression analysis in *Xenopus* showed that also Lrp5, another co-receptor in canonical Wnt signaling [15], is expressed in the neural crest and its derivatives [16]. In mammals, Lrp5 plays a major role in bone homeostasis, and mutations in *LRP5* are associated with reduced bone mass leading to the osteoporosis-pseudoglioma syndrome in humans [17]. Conversely, gain of function mutations in *LRP5* at the N-terminus lead to a high bone mass phenotype as binding of its endogenous inhibitor Sost is prevented [18–20]. Mutations in *Lrp5* in mice lead to reduced proliferation of osteoblast precursors [21]. On the other hand, patients with loss of function mutations in *SOST* suffer from sclerosteosis, a progressive sclerosing bone dysplasia, comparable to gain of function mutations of *LRP5* in humans [22,23]. So far, no direct links between mutations in *LRP5* and early developmental defects of the craniofacial skeleton have been made in mammals. Importantly, however, there are reports about cranial bone dysmorphologies in human patients with *LRP5* gain of function mutations, such as craniosynostosis at an early age [24] or a large lobulated torus palatinus and an abnormally thick mandibular ramus [25]. This opens the possibility that a potential role of *lrp5* in craniofacial morphogenesis is conserved between different groups of vertebrates.

In the present study, we analyzed *lrp5* expression during zebrafish embryogenesis and used gene knock-down as well as transient CRISPR/Cas9 mediated gene editing to study its function during head skeleton formation. We show that zebrafish *lrp5* is ubiquitously expressed at early stages but gets restricted to CNCC derivatives during later embryonic and larval development. Interfering with *lrp5* function results in reduced proliferation of neuroepithelial cells in the hindbrain causing migration defects of CNCCs, which ultimately result in severe developmental defects of the cranial skeleton. Our data thus are consistent with a model that proposes a link between cell cycle progression and NCC delamination [12] and assigns an important role for Lrp5 in this process.

## Material and Methods

### Fish keeping and husbandry

The authors confirm that the Institutional Animal Care and Use Committee (IACUC) of the National University of Singapore (NUS) has specifically approved this study (approved protocol numbers 020/08, BR19/10, 014/11). Adult zebrafish of DBS inbred wild-type strain as well as *sox10*:GFP [26], *fli1*:EGFP [27] and TOPdGFP transgenic zebrafish [28] were used to obtain embryos that were raised in 30% Danieau’s solution at 28°C and staged as described previously [29]. At desired stages, embryos and larvae were fixed in 4% paraformaldehyde (PFA).

### Sequence characterization

Protein sequences of Lrp5 in zebrafish (Lrp5_Dr; ENSDARG00000006921), human (Lrp5_Hs; ENSG00000162337), mouse (Lrp5_Mm; ENSMUSG00000024913), chicken (Lrp5_Gg; ENSGALP00000011316), *Xenopus* (Lrp5_Xt; ENSXETG00000010024), Fugu (Lrp5_Tr; ENSTRUP00000006121), medaka (Lrp5_Ol; ENSORLG00000018545) and *Drosophila* (Arrow_Dm; FBgn0000119) were retrieved from Ensembl (ensemble.org) and compared using ClustalW [30]. Pairwise sequence alignments were generated with bl2seq [31].

### Generation of a *lrp5* riboprobe and *in situ* hybridization

For preparation of a *lrp5* riboprobe (ENSDARG00000006921), total RNA isolated from zebrafish embryos at 3 days post fertilization (dpf) was reverse transcribed and primers Lrp5up1 (CCATCAAACAGACCTACTACAACCT) and Lrp5down1 (GAATATCATTGACTTGAAGGACGAT) were used to amplify a 885 bp fragment. Similarly, a *crestin* antisense probe template was generated using primers crestinup (GCCAAGATGTTCACGCCTAT) and crestindown (GTTGCATCAAGGTGGTGTTG). For generation of Digoxigenin (DIG)-labeled riboprobes, the following restriction enzyme and polymerase combinations were used: for *lrp5*, X*hoI*, T7 RNA-polymerase; *crestin*, B*amHI*, Sp6; *gfp*, H*indIII*, T7; *lef1*, N*otI*, SP6; *ccnd*, B*amHI*, T7; *dlx2a*, B*amHI*, T7; *foxd3*, B*amHI* T7. In situ hybridizations were performed as described previously [32] except that duration of proteinase K digestion was adjusted to the used embryonic and larval stages.

### Morpholino and CRISPR/Cas9 injection

For gene knock-down of *lrp5*, the following splice blocking Morpholinos (MOs; Gene Tools, Corvalis, USA) were used: *lrp5*MoUp (AGCTGCTCTTACAGTTTGTAGAGAG) targets the Exon2-Intron2 splice junction; *lrp5*MoDown (CCTCCTTCATAGCTGCAAAAACAAG) targets the Intron2-Exon3 splice junction (see S1 Fig). A mismatch morpholino (mm*lrp5*) containing five nucleotide substitutions (AGgTGCTgTTAgAGTTTcTAGAcAG) was designed as control. The used p53-Morpholino was previously described [33]. For injection, 3 mM stock solutions were diluted with H_2_O and 0.1% phenol red was added. Working solutions were injected into the yolk of one or two-cell stage embryos as described previously [34]. To assess knock-down efficiency by semi-quantitative RT-PCR, total RNA was isolated from wild-type and MO injected embryos at the 25 somite stage (ss). After DNase treatment and reverse transcription, spliced transcripts were PCR amplified with primers Lrp5MoChkup (CAGTGGACTTTCTCTTCTCG) and Lrp5MoChkdown (GTCTCCGAGTCAGTCCAGTA). To amplify transcripts with retained introns, primers Lrp5MoChkup and Lrp5MointronChkdown (CTAAGATTGTGGGTCACAGG) were used.

Two *lrp5* CRISPR guide RNAs were designed using ZiFiT (http://zifit.partners.org/ZiFiT/). CRISPR1 targets exon 2 of *lrp5* (target sequence: TCTGGAGGACGCGGCCGCAG), while CRISPR2 targets exon 3 (GGTGCTCTTCTGGCAAGATC). *Cas9* mRNA was synthesized from pCS2-nCas9n (Addgene #47929) [35] using SP6 RNA polymerase after *Not*I linearization. The guide RNAs were injected individually (200 pg) or in combination (200 pg each) together with 300 pg of *cas9* mRNA into the cytoplasm of one-cell staged embryos. Restriction fragment length polymorphism (RFLP) analysis was done to detect mutagenic events. For *lrp5* CRISPR1, a 168bp fragment was amplified using primers 5’-GTCTCCTTTGCTGCTTTTCG-3’ and 5’-GGTCTGTTTGATGGCCTCCT-3’ and digested with *Not*I to result in 102bp and 66bp fragments when the target site was not mutated. For *lrp5* CRISPR2, primers 5’-GTGGTGGTTTCAGGTCTGGA-3’ and 5’-GAGAGGGG TTTAGTGCAATCG-3’ were used and a 181bp fragment was digested with *Bgl*II to result in 141bp and 40bp products when no mutation occurred. PCR and digestion products were analysed on a 1.5% TAE agarose gel for genotyping.

### Immunostaining and skeletal staining

Immunohistochemistry was carried out as previously described [34]. To stain for cells in M-phase, rabbit derived monoclonal anti-phosho-histone3 (pH3) antibody (Upstate Biotechnology, NY) was used in combination with anti-rabbit Alexa568 coupled secondary antibody (Invitrogen). To detect cells in S-phase, embryos were incubated in 10 mM BrdU for 30 minutes, washed several times and kept another 30 minutes before fixation in 4% PFA overnight. After this, embryos were washed in PBST and kept overnight in methanol. Then, embryos were rehydrated, followed by incubation in 2N HCl for 1 hour at 37°C. BrdU-positive nuclei were stained by using mouse anti-BrdU antibody (BSHB, Iowa City, IA; diluted 1:500 in PBDT) in combination with anti-mouse Alexa 488 coupled secondary antibody (diluted 1:1000; Invitrogen). Combined staining for cartilage (Alcian Blue) and bone (Alizarin Red) was performed as previously described [36].

### Image acquisition

For microscopy, stained embryos were mounted in 100% glycerol. For flat-mount preparations, the yolk was manually removed. Images were taken with a Nikon SMZ1000 stereomicroscope, a Nikon T1-SM inverted microscope with GFP filter set and a Nikon Eclipse 90i upright microscope using the NIS-element BR software (Nikon). For confocal microscopy, a LSM 510 Meta laser scanning confocal microscope (Zeiss) was used. Alexa488 was detected by excitation with an argon multi-line gas laser at 488 nm and detection through the BP 505–530 nm filter. Alexa568 was detected by excitation with a Helium Neon gas laser at 543 nm and detection through the LP 560nm filter. LSM software (Zeiss) was employed for confocal image processing. For histological analysis, specimen were fixed in a mixture of 1.5% glutaraldehyde and 1.5% paraformaldehyde in 0.1 M cacodylate buffer and processed for embedding in Epon, according to standard procedures. Transverse 1 μm semi-thin cross sections of the head were stained with toluidine blue for 1–2 min (0.2% toluidine blue, 2% Na2CO3), rinsed with water, air-dried and mounted with DPX (Fluka, Buchs, Switzerland). A Zeiss Axio Imager—Z1 compound microscope equipped with a Zeiss Axiocam MRc camera was used for imaging.

### Quantification and statistical analysis

For analysis of cell proliferation, cell numbers were determined in images of multiple individuals (n). A region of interest (ROI) was defined to cover the hindbrain region between rhombomeres 4 to 8 (r4-8). Within this area, all positively stained nuclei were counted. Average, standard deviation and *P*-values (Student’s T-test) were determined using Microsoft Excel 2011.

## Results

### Characterization of the zebrafish *lrp5* sequence

**Fig 1:**
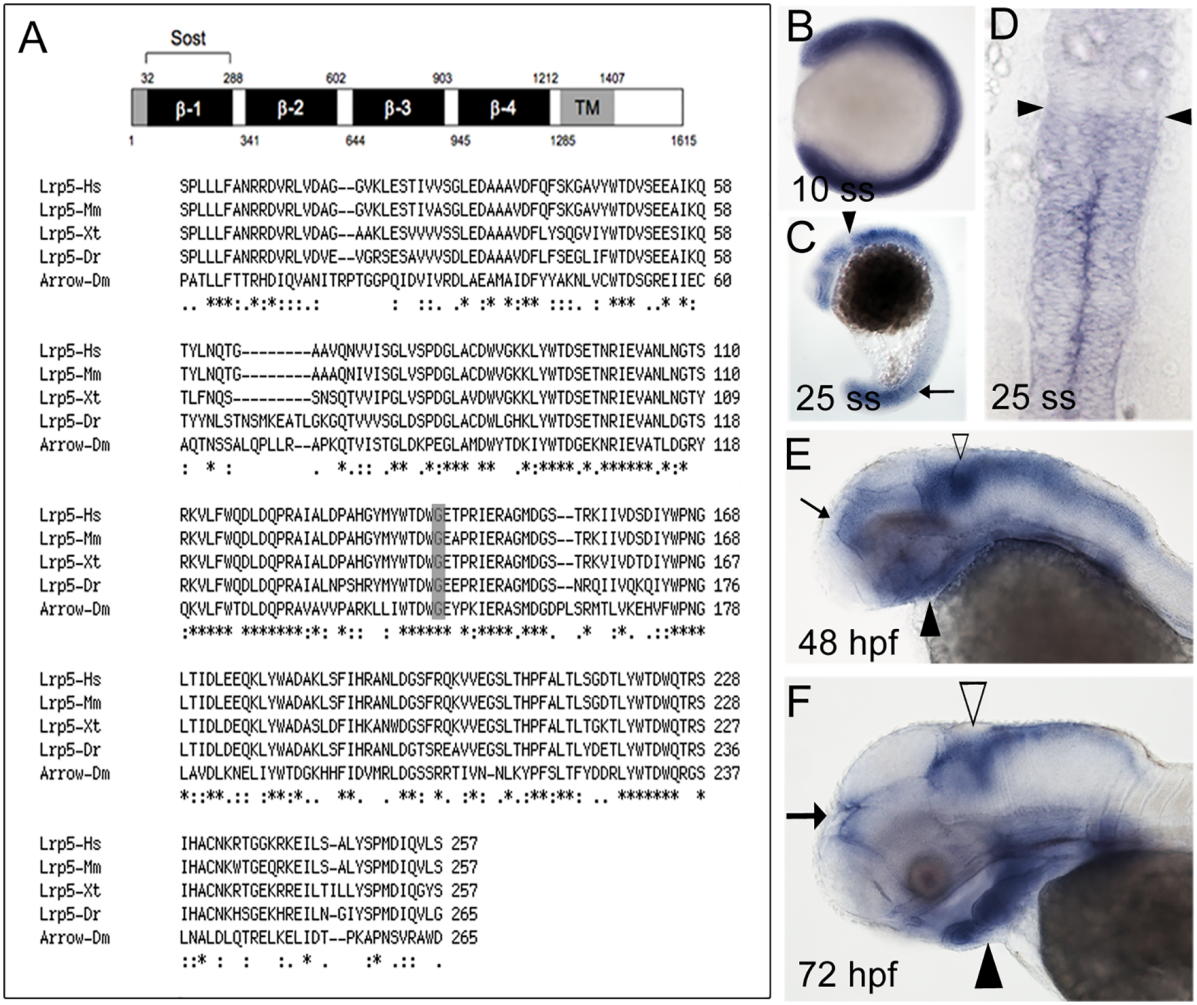
Lrp5 sequence alignment and expression pattern. (**A**) Schematic illustration of predicted Lrp5 protein domains (top). Numbers indicate amino acid positions and refer to human Lrp5. Grey boxes represent signal peptide (1–32) and transmembrane domain (TM), respectively. β-1 to β-4 indicate β-propeller domains 1 to 4. The β-1 domain is proposed to bind to Sost. Bottom: Alignment of amino acid sequences in the β-1 domain. Glycine at position 171, which is mutated to valine in human patients with high bone mass phenotypes [25] is highlighted in grey. (**B-F**) Spatiotemportal expression of *lrp5* during embryonic and larval development: Expression at 10 ss (B), 25 ss (C,D), 48 hpf (E) and 72 hpf (F). Anterior is to the left in B,C,E,F and to the top in D.

Sequence information for zebrafish *lrp5* is available in Ensembl (ENSDARG00000006921). *Lrp5* is located on chromosome 25 and spans a region of more than 120 kb (10,854,422–10,981,755). It contains an open reading frame (orf) of 4845 base pairs (bp) in 24 exons. The deduced amino acid (aa) sequence is 1615 aa. Sequence comparisons revealed identities of 76% (to human); 75% (mouse); 79% (chicken); 76% (Xenopus); 67% (Fugu); 80% (Medaka) and 45% (Drosophila), respectively. Thus, the degree of conservation of the Lrp5 amino acid (aa) sequence is high. Fig 1A shows an aa sequence alignment for the first YWTD propeller motif (β1). This motif is an important regulatory domain and is the binding site for SOST [37]. A glycine residue at position 171, which is mutated to valine in human patients with high bone mass phenotypes [25], is conserved in all analyzed species including zebrafish (highlighted in grey in Fig 1A).

### *lrp5* expression during zebrafish embryogenesis

In zebrafish, *lrp5* is maternally expressed and transcripts are found throughout the embryo at the eight-cell stage (data not shown). *lrp5* remains ubiquitously expressed through gastrulation until the 10 somite stage (ss) (Fig 1B). Around 25 ss, its expression becomes restricted with elevated levels found in the developing brain and tail (arrow in Fig 1C). In the brain, regionally restricted expression is found in the fore-, mid- and hindbrain with the anterior hindbrain region being devoid of any expression (arrowheads in Fig 1C and 1D). At 48 hpf, strong *lrp5* expression persists in the head region (Fig 1E) and weaker expression is observed in the trunk (not shown). Broad expression is found throughout the head with elevated levels in the dorsal hindbrain (white arrowhead in Fig 1E) and the diencephalon (arrow in Fig 1E). In contrast, expression is weak or absent in tectum and telencephalon. *lrp5* is also strongly expressed in the ventral head region (black arrowhead in Fig 1E). At 72 hpf, *lrp5* expression becomes further restricted to distinct domains (Fig 1F). It is strongly expressed in the cranial region as well as in the pectoral fin anlagen (not shown). In the head, expression is found predominantly in the dorsal hindbrain (white arrowhead in Fig 1F) including the rhombic lip, as well as epiphysis (arrow, Fig 1F). No expression is detectable in tectum and telencephalon but strong expression is found throughout the forming pharyngeal skeleton in the ventral head region (black arrowhead, Fig 1F), which contains CNCC derivatives.

### *lrp5* knock-down leads to defects in hindbrain and CNCCs

To analyze the role of *lrp5* during zebrafish embryogenesis, we first used a Morpholino (MO) based knock-down approach. Two separate splice blocking MOs (*lrp5*MoUp; *lrp5*MoDown, Figure A in S1 Fig) targeting intron 2–3 were injected alone or in combination at different concentrations and tested for efficacy. All injections resulted in similar phenotypes but with different degrees of severity depending on the concentration used and whether MOs were injected alone or in combination (Figures C and D in S1 Fig). The most severe phenotypes were obtained by a combination of 0.3 mM *lrp5*MoUp and 0.3 mM *lrp5*MoDown. This setting was used for all experiments described below and henceforth addressed as *lrp5*Mo. Separate injection of each splice MOs resulted in identical phenotypes supporting specificity of the obtained phenotype and excluding the possibility that they are caused by unspecific off-target effects (Figures C and D in S1 Fig). A mismatch control MO (*lrp5 mm*MO) did not lead to obvious morphological defects (Figures C and D in S1 Fig). To determine the efficiency of the MO knock-down, semi-quantitative RT-PCR was performed on injected embryos. This showed a clear reduction of correctly spliced *lrp5* cDNA in *lrp5* morphants compared to wild-type and mismatch control morphants (Figure B in S1 Fig). In addition, occurrence of a second band suggested an alternatively spliced product in morphant cDNA. When intron retention was analyzed, the amount of non-spliced transcript was significantly higher in *lrp5* morphants compared to wild-type and *mm*MO injected embryos (Figure B in S1 Fig). β*-actin* transcript levels were not significantly altered. Thus, injection of a combination of splice blocking MOs resulted in a considerable knock-down of *lrp5*.

**Fig 2:**
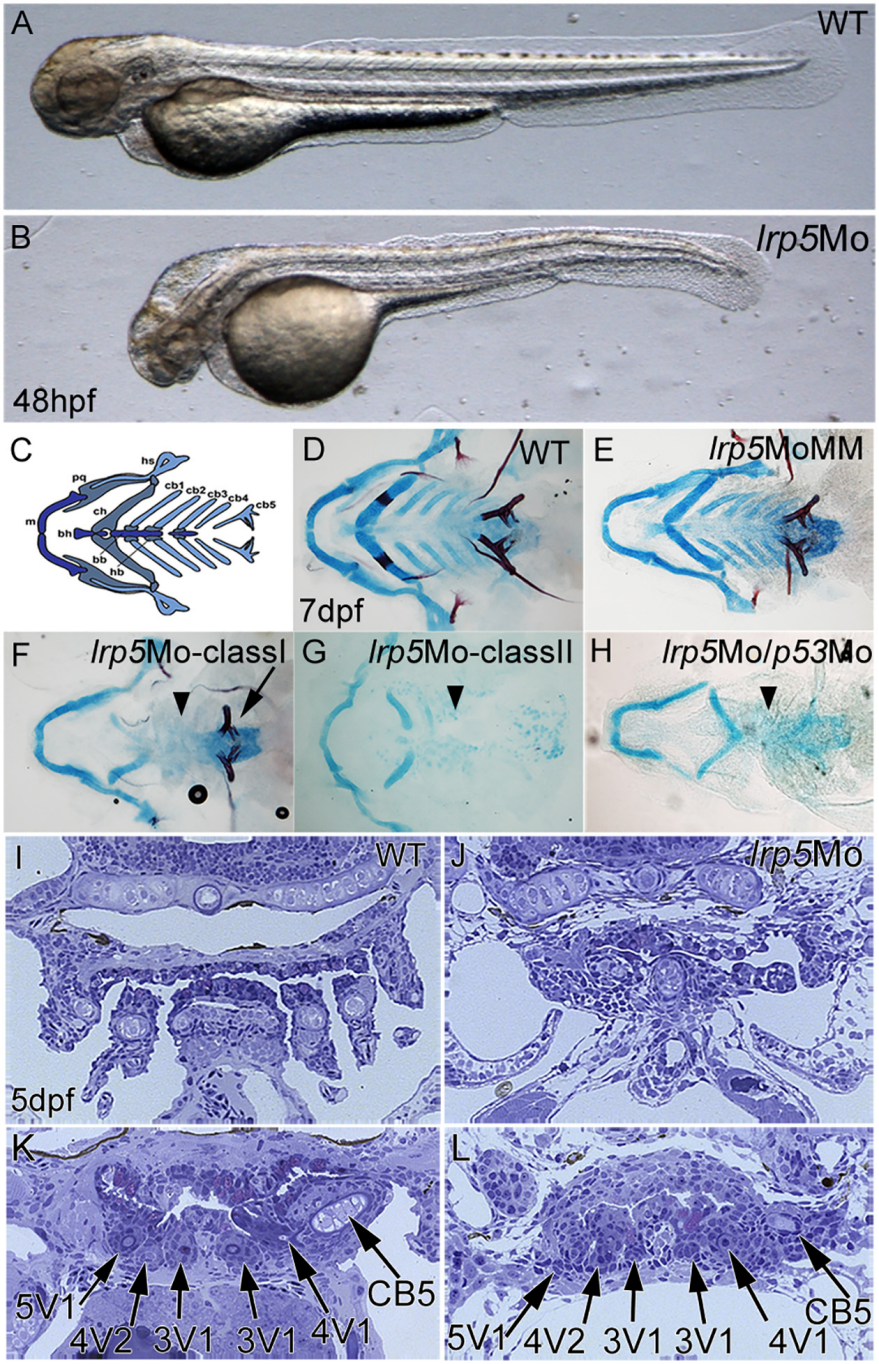
Knock-down of *lrp5* leads to defects in the craniofacial skeleton but not teeth. (**A,B**) Morphology of wild-type and *lrp5* morphant embryos at 48 hpf. Note inflated hindbrain in morphant. (**C**) Schematic illustration of viscerocranial skeleton formed mainly by CNCCs (different colors represent different groups of skeletal elements; bb, basibranchial; bh, basihyal; cb, ceratobranchial; ch, ceratohyal; hb, hypobranchial; hs, hyosymplectic; m, Meckel’s cartilage; pq, palatoquadrate). (**D-H**) Combined bone and cartilage staining at 7 dpf of wild-type (D), *lrp5*MM morphant (E), *lrp5* morphant classI (F), classII (G) and *lrp5/p53* compound morphant (H). Note that morphants show absence of ceratobranchials (arrowheads) while 5^th^ ceratobranchial and pharyngeal teeth (arrow) are present in classI morphants. (**I-L**) Cross sections through 5 dpf larvae. Wild-type (I) shows ceratobranchials, which are lost in *lrp5* morphant (J). More posterior sections show that wild-type (K) and *lrp5* morphants (L) have normally formed pharyngeal teeth (arrows). Anterior is to the left in A-H.

Consistent with an earlier report [38], knock-down of *lrp5* resulted in severe hindbrain defects in embryos, which were morphologically most obvious at 48 hpf. Compared to wild-type controls (Fig 2A), *lrp5* morphants had widely inflated hindbrain ventricles (Fig 2B). CNCCs originate from the dorsal hindbrain, migrate ventrally and form large parts of the cranial skeleton (Fig 2C). We checked for the morphology of the ventral cranial skeleton in *lrp5* morphants at larval stages by bone and cartilage staining. Compared to wild-type (Fig 2D) and MoMM injected embryos (Fig 2E), *lrp5* morphants exhibited severe malformations of the cranial skeleton (Fig 2F and 2G). In *lrp5 mm*MO injected embryos, general development was slightly delayed as evident by delayed mineralization of the ceratohyals (compare Alizarin red staining in Fig 2D and 2E). Importantly however, CNCC derived cartilage structures formed normally in *lrp5 mm*MO injected embryos (Fig 2E). In *lrp5* morphants, cranial phenotypes were grouped into two classes of severity. ClassI morphants were characterized by a complete loss of ceratobranchials 1–4 (arrowhead in Fig 2F) and reverse oriented ceratohyals. The 5^th^ ceratobranchial with attached pharyngeal teeth appeared normal (arrow in Fig 2F). In more severely affected classII morphants, only rudiments of the ventral craniofacial skeleton such as Meckel’s cartilage or ceratohyal remained while the 1^st^ to 5^th^ ceratobranchials were completely absent (arrowhead in Fig 2G). Importantly, these defects could not be rescued by co-injection of a *p53* MO (Fig 2H), excluding the possibility of unspecific apoptosis caused by MO off-target effects.

Analysis of toluidin blue stained histological sections of classI morphant larvae at 5 dpf revealed that pharyngeal cartilages were present and well differentiated in some areas, however their number, shape and position was affected (Fig 2J). This made their identification difficult with exception of the hyosymplectic. Wild-type larvae on the other hand showed well differentiated cartilaginous arches at this stage (Fig 2I). Interestingly, dentition progressed normally in *lrp5* morphants despite severe skeletal malformations. According to [39], wild-type larvae at 5 dpf (Fig 2K) possess three teeth on each side, labeled 3V^1^, 4V^1^ and 5V^1^, with 4V^1^ attached and possessing a replacement tooth, 4V^2^, in early cytodifferentiation. Teeth 3V^1^ and 5V^1^ are in a similar stage at late cytodifferentiation and do not have a replacement tooth yet. In *lrp5* morphants (Fig 1L), the same three teeth are present: 3V^1^, 4V^1^ and 5V^1^. Individuals with tooth 4V^1^ in late cytodifferentiation have teeth 3V^1^ and 5V^1^ in the morphogenesis stage. Individuals with tooth 4V^1^ in an early cytodifferentiation stage have teeth 3V^1^ and 5V^1^ in an initiation stage only. Tooth 4V^1^ displays no replacement tooth, but this is to be expected given that a replacement tooth develops only once its predecessor is attached, which is not the case. Taken together, analyzing tooth organization in *lrp5* morphant larvae revealed that not all ventral head structures are generally affected. Rather, tooth development as well as development of other dermal skeletal elements, such as cleitra and operculae, appeared normal (or at most slightly delayed), while CNCC derived cartilage elements of the head skeleton were strongly affected. This opens the possibility that *lrp5* at this stage is required for morphogenesis of specific CNCC derived craniofacial cartilage structures but not for head development in general.

### Knock-down of *lrp5* reduces canonical Wnt signaling

**Fig 3:**
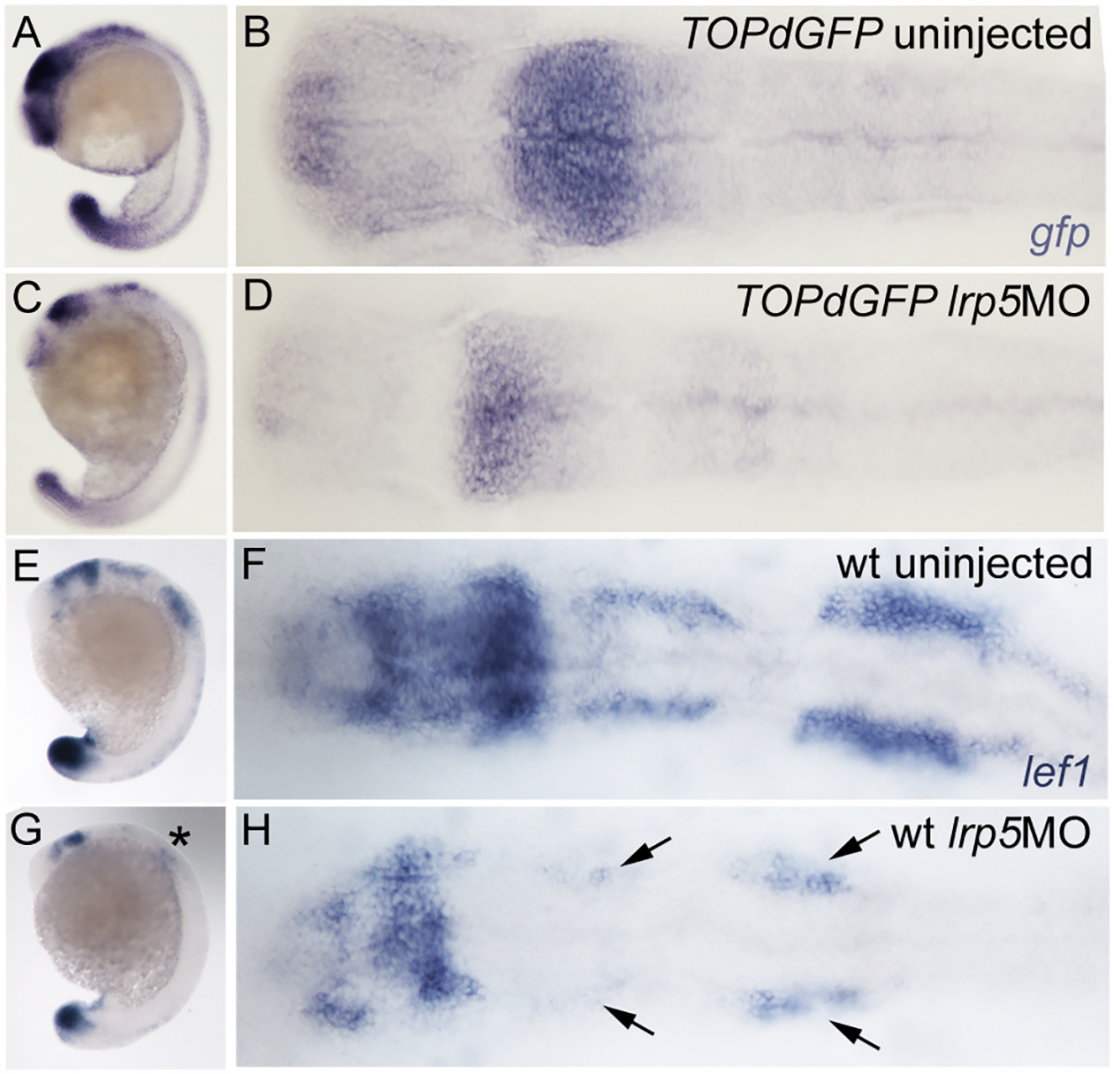
Knock-down of *lrp5* results in reduced canonical Wnt signaling. (**A-D**) TOPdGFP embryos at 20 ss analyzed for *gfp* transcription. (A,B) Uninjected control, (C,D) *lrp5* morphant. Note that *gfp* transcripts are down-regulated in morphants. (**E-H**) 20 ss embryos expressing *lef1*. (E,F) Wild-type embryo, (G,H) *lrp5* morphant. Note that *lef1* expression is down-regulated in morphants, particularly in the CNCC regions (see asterisk in G and arrows in H). Anterior is to the left in all images.

We next tested whether a *lrp5* knock-down leads to reduced Wnt activity, in particular in the hindbrain [38]. For this, *lrp5*MOs were injected into TOPdGFP transgenic zebrafish embryos expressing destabilized GFP under control of a Lef1/β-catenin responsive element [40]. At 20 ss, all analyzed control embryos (n = 33) showed strong reporter activity in the mid-hindbrain boundary (MHB), as well as the hindbrain and tailbud as analyzed by *in situ* hybridization with a *gfp* riboprobe (Fig 3A and 3B) [40]. In *lrp5* morphants, however, 80% (n = 51) of embryos showed an overall decrease of GFP expression including in the MHB and hindbrain (Fig 3C and 3D). This suggests a drastic down regulation of Wnt signaling after injection of *lrp5* MOs.

To test whether Wnt signaling is reduced specifically in neural crest cells, we examined expression of *lymphoid enhancer-binding factor 1* (*lef1*), a key downstream factor in Wnt signal transduction [41]. *lef1* is expressed in migratory CNCCs at 20 ss as two bilateral stripes adjacent to the hindbrain (Fig 3E and 3F) [42]. 73% (n = 80) of *lrp5* morphants showed a clear reduction in *lef1* expression in all expression domains including the CNCCs (asterisk in Fig 3G, arrows in Fig 3H).

### Interfering with *lrp5* function affects migration, but not induction of CNCCs

**Fig 4:**
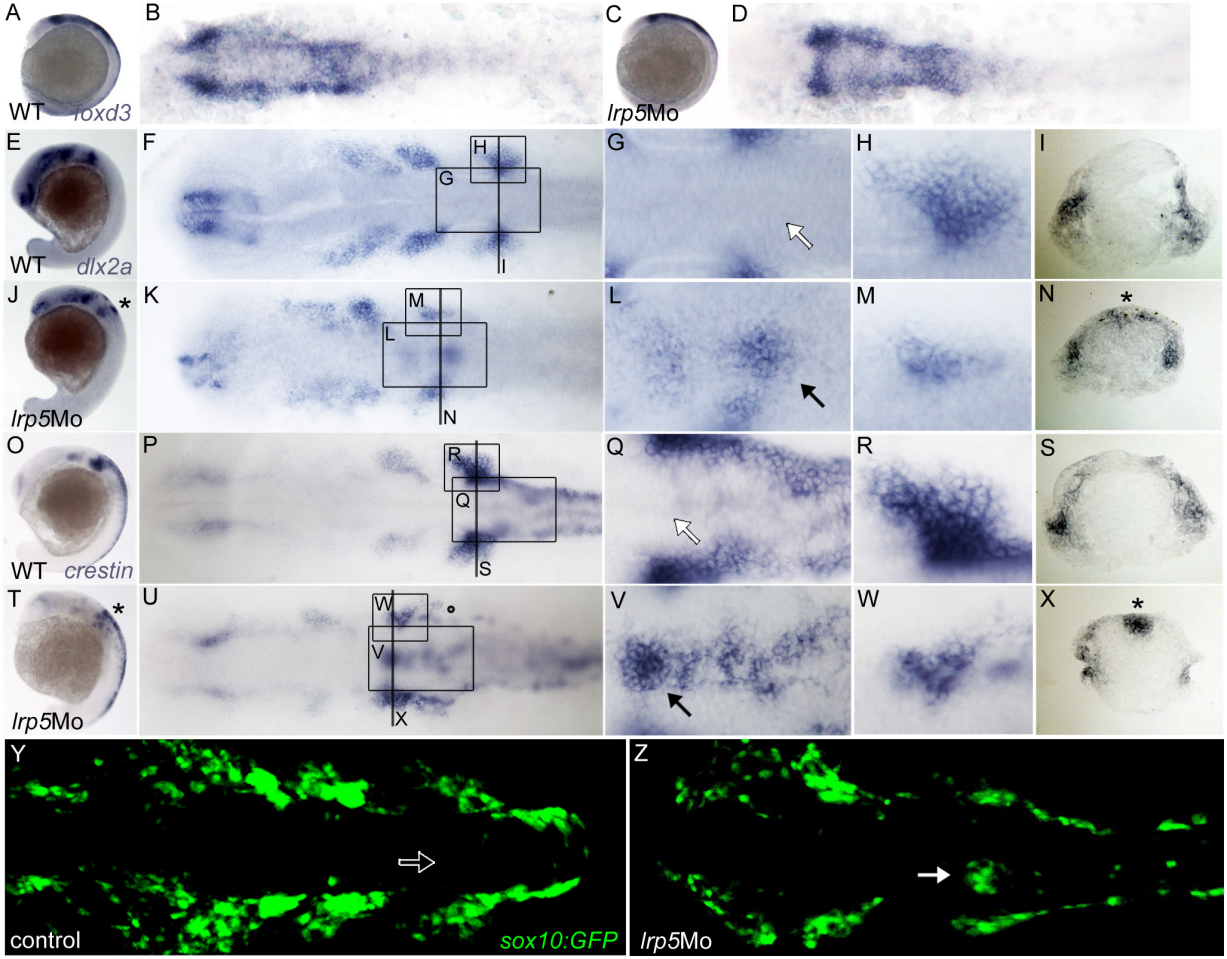
*lrp5* morphants display normal induction but defective migration of CNCCs. (**A-D**) Embryos at 10 ss stained for *foxd3* transcripts. (A,B) Wild-type embryo, (C,D) *lrp5* morphant. Note normal pattern of *foxd3* expression in morphants. (**E-N**) Embryos at 20 ss stained for *dlx2a*. (E-I) Wild-type embryo, (J-N) *lrp5* morphant. Note ectopic *dlx2a* expression at dorsal neuroepithelium of rhombomere 6 in *lrp5* morphants (asterisk in J,N; arrow in L) and that streams of branchial migratory CNCCs are reduced (M). (**O-X**) *crestin* expression in embryos at 20 ss. (O-S) Wild-type embryo, (T-X) *lrp5* morphant. Note ectopic *crestin* expression at dorsal neuroepithelium of rhombomere 6 in *lrp5* morphants (asterisk in T,X; arrow in V) and that streams of branchial migratory CNCCs are reduced (M). (**Y,Z**) Confocal projections of *sox10*:GFP embryos at 20 ss showing GFP expression in CNCCs. (Y) Uninjected control embryo, (Z) *lrp5* morphant. Note ectopic GFP cells at dorsal neuroepithelium of rhombomere 6 in *lrp5* morphants (arrow) and that streams of branchial migratory CNCCs are reduced. Anterior is to the left in all images, except cross sections. Boxed areas indicate regions shown in higher magnification in accompanying images. Positions of cross sections in I,N,S,X are indicated by lines in F,K,P,U.

Wnt signaling has been shown to play multiple roles in neural crest induction, migration and differentiation [10]. To examine whether *lrp5* is required for neural crest induction, we analyzed expression of the early CNCC marker *forkhead box d3* (*foxd3* or *fkd6*) [43]. At 10 ss, *foxd3* expressing premigratory CNCCs are found bilaterally to and overlaying the dorsal neuroepithelium in the caudal hindbrain (Fig 4A and 4B). Expression of *foxd3* was normal in *lrp5* morphants (100%, n = 28; Fig 4C and 4D) when compared to wild-type controls (n = 32; Fig 4A and 4B). This suggests that *lrp5* is not involved in early CNCC induction.

Next, we analyzed whether CNCC migration is altered after *lrp5* knock-down. At 14–15 hpf, CNCCs start to migrate from positions lateral to rhombomeres 2, 4 and 6 towards the ventral pharyngeal arch region in three distinct streams. At 20 ss, cells in these streams express *distal-less homeobox 2a* (*dlx2a*; Fig 4E–4I) [44]. No *dlx2a* expression is found in the medial neuroepithelium (arrow, Fig 4G; transverse section, I). Knock-down of *lrp5* drastically changed the pattern of migratory CNCCs. First, all CNCC streams appeared strongly reduced and disorganized in 45% of the *lrp5* morphants (n = 64; Fig 4J and 4K). Moreover, a patch of ectopic *dlx2a* positive cells was observed in a dorsomedial position above the neural tube between the branchial CNCC streams at r6 (asterisks and arrow, Fig 4J,4K,4L and 4N). The r6 stream was severely reduced in size (Fig 4M) when compared to wild-type controls (Fig 4H). No changes in pattern were evident in *lrp5 mm*Mo injected embryos (n = 59; Figures G and H in S1 Fig). This suggests that CNCC migration in the branchial stream is disrupted upon reduced Lrp5 activity.

To confirm this, migratory CNCCs were also examined for expression of the pan-neural crest marker *crestin* [45]. In wild-type embryos, *crestin* is expressed in cranial and trunk neural crest cells (Fig 4O). In controls at 20 ss, no *crestin* positive cells are found in the region dorsal to the neuroepithelium (arrow, Fig 4Q) but in two bilateral streams of migratory NCCs (Fig 4P,4Q and 4R; transverse section in S). In 55% (n = 64) of *lrp5* morphants, however, clusters of ectopic *crestin* positive NCCs were found in dorsomedial positions comparable to the situation for *dlx2a* (arrow, Fig 4V; asterisk in transverse section X). Likewise, branchial clusters of *crestin* positive migratory CNCCs were drastically reduced in size (Fig 4W) when compared to wild-type (Fig 4R) or MM morphants (n = 54; Figures K and L in S1 Fig).

To obtain further evidence for migratory defects in *lrp5* morphants, *lrp5*MO was injected into *sox10*:GFP transgenic embryos [26]. In this line, cells of the neural crest lineage express GFP in migratory streams around 20 ss, and no GFP-positive cells are found in the dorsal hindbrain (arrow in Fig 4Y). In contrast, 54% of *sox10*:GFP embryos injected with *lrp5*Mo (n = 74) showed ectopic clusters of GFP positive cells in dorsomedial positions (arrow in Fig 4Z) similar to the situation for *dlx2a* and *crestin*. Also in *lrp5* deficient *sox10*:GFP embryos, caudal clusters of migratory CNCCs were smaller when compared to controls.

Taken together, this suggests that a knock-down of *lrp5* results in altered migratory behavior of CNCCs, while induction is not affected. Although the observed ectopic cells have migratory CNCC character as evident by *dlx2a* expression, they fail to follow the migratory streams and instead are retained in dorsomedial positions [46].

**Fig 5:**
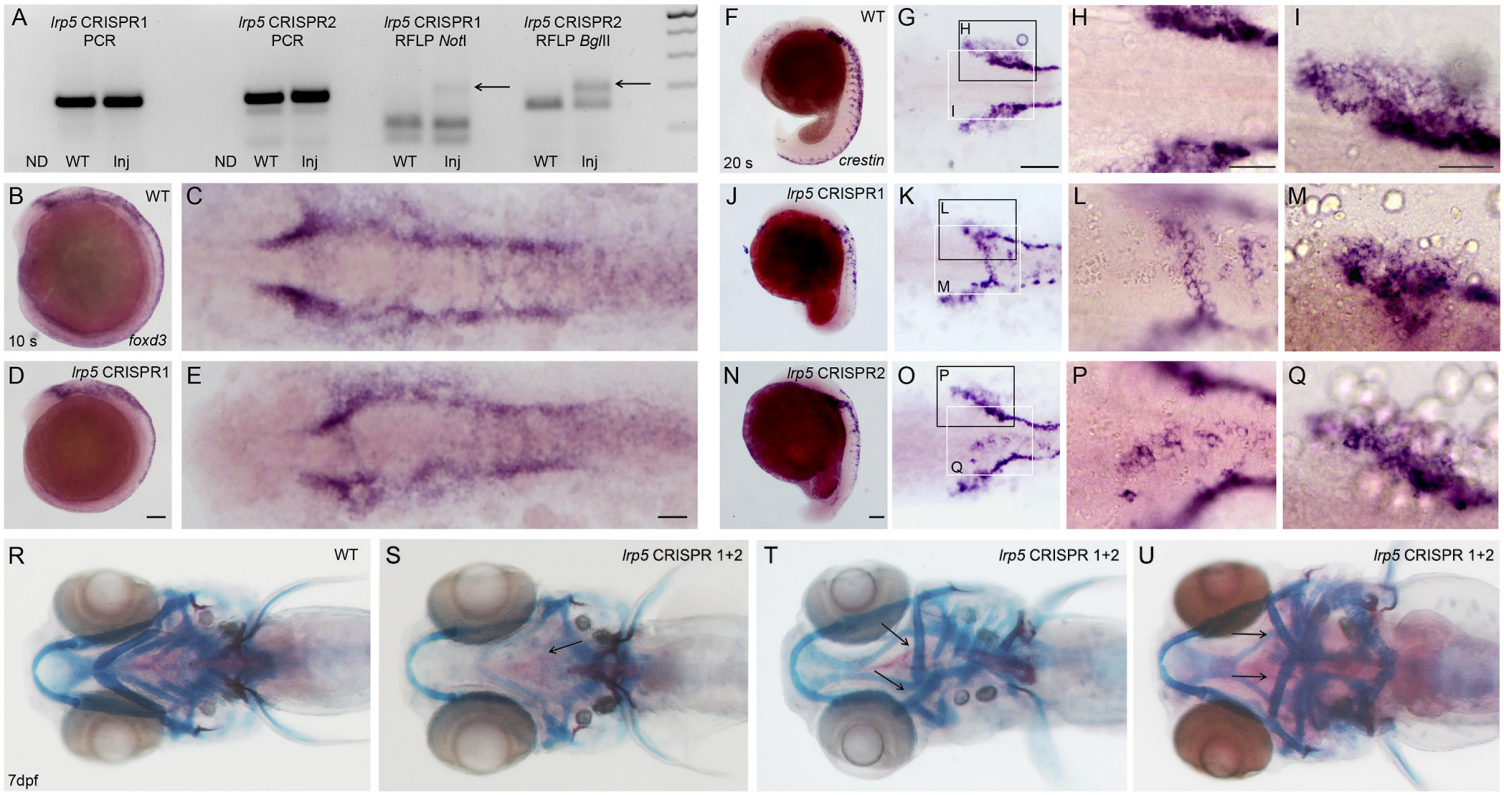
*lrp5* CRISPR/Cas9 injected embryos display normal CNCC induction, but defective CNCC cell migration. (A) RFLP analysis of 15 pooled embryos per sample. Both, *lrp5* CRISPR1 and CRISPR2 generate mutations indicated by presence of undigested mutant bands after *Not*I and *Bgl*II digestion (right lanes), respectively. Left lanes show non-digested (ND) PCR products. (B-E) In situ hybridization showing *foxd3* expression at 10 ss in wild-type embryos (B, C) and *lrp5* CRISPR1 injected embryos (D,E). (F-Q) *crestin* expression at 20 ss in wild-type embryos (F-I), *lrp5* CRISPR1 injected embryos (J-M), and *lrp5* CRISPR2 injected embryos (N-Q). (R-U) Combined bone and cartilage staining at 7 dpf in wild-type (R) and *lrp5* CRISPR1 and CRISPR2 co-injected embryos showing cartilage defects with different degrees of severity. Note absence of ceratobranchials 1–4 (in S; arrow), and flipped ceratohyal (in T,U; arrows). (B,D,F,J,N) Lateral views with anterior to the left. All remaining images are dorsal views, except (R-U), which are ventral views. Higher magnification views of areas boxed in (G,K,O) are shown in accompanying images (I, M and Q, white boxes) and (H, L and P, black boxes). Note ectopic *crestin* positive cells in dorsal neuroepithelium (K,L,O,P) and reduced migratory CNCCs streams (K,M,O,Q) in *lrp5* CRISPR injected embryos. Scale bars: 100 μm (D,G,N) and 50μm (E,H,I).

To validate the MO induced phenotypes, a CRISPR/Cas9 approach was used and two guide RNAs were designed and injected separately in a transient gene targeting assay. RFLP analysis revealed that both guide RNAs were efficient in mutating the chosen target sequence (Fig 5A). Separate injections of both guide RNAs did not affect expression of *foxd3* (n = 43, Fig 5B–5E; and Figures A to D in S2 Fig) indicating that the induced mutations did not alter neural crest induction. On the other hand, both guide RNAs resulted in ectopic *crestin* positive cells in dorsomedial positions in a distinct fraction of embryos (n = 8/38 for *lrp5* CRISPR1 and 14/47 for CRISPR2, respectively; Fig 5F–5Q), thus recapitulating the situation in *lrp5* MO injected embryos. *lrp5* CRISPR/Cas9 injected embryos also developed the typical severe defects in the ventral craniofacial skeleton (Fig 5R–5U), recapitulating the phenotypes seen in *lrp5* morphants. Together this shows that the two used CRISPR/Cas9 guide RNAs validate the phenotypes observed after *lrp5* knock-down and therefore confirms that *lrp5* is required for neural crest cell migration and branchial arch formation.

### A knock-down of *lrp5* leads to reduced proliferation of premigratory CNCCs

**Fig 6:**
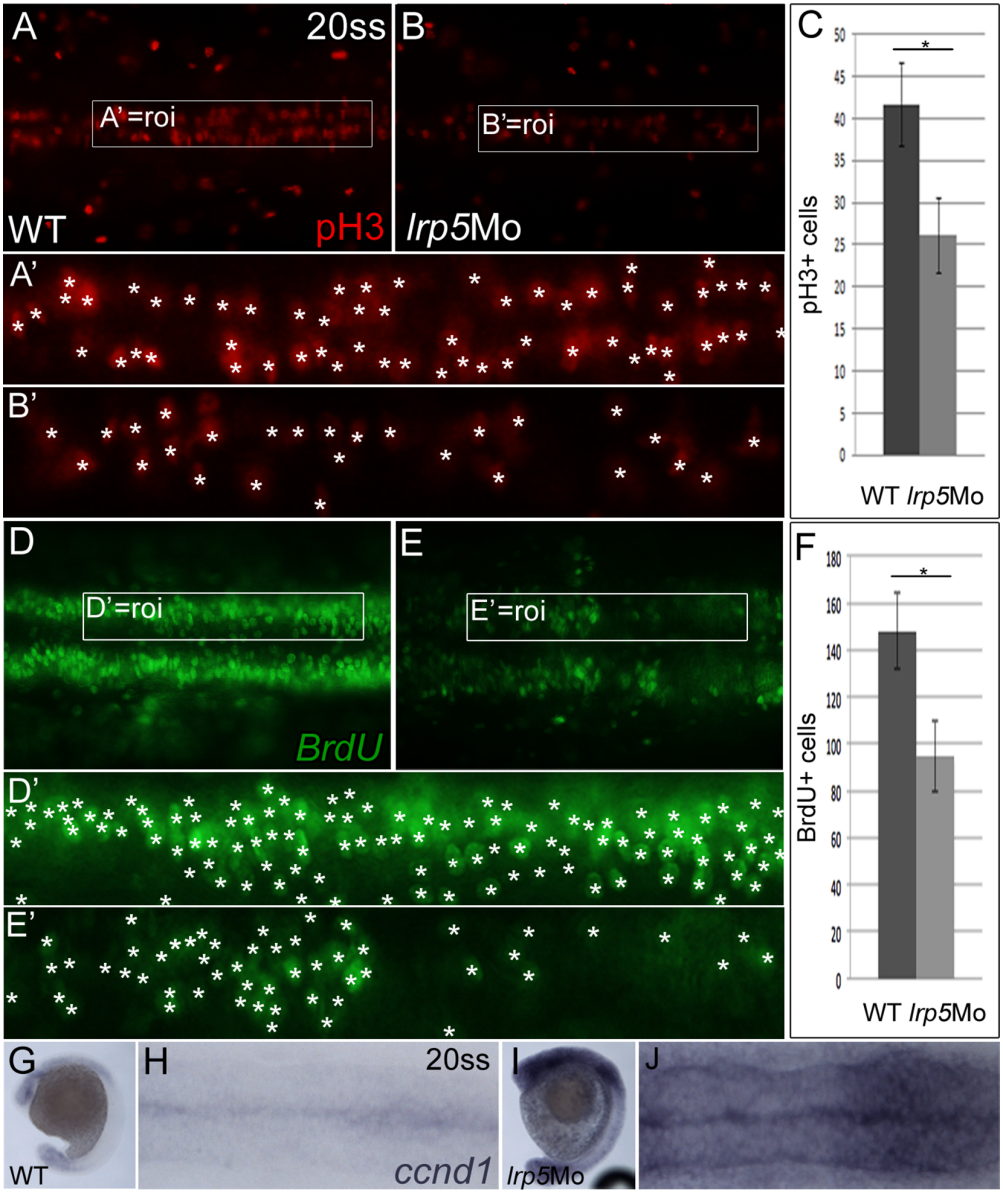
Proliferation of premigratory CNCCs is affected by knock-down of *lrp5*. (**A,B** *)* 20 ss embryos stained for pH3 cells in M-phase. (A) Wild-type embryo, (B) *lrp5* morphant. Frames demarcate area of cell count (roi, region of interest) and are magnified in (**A’,B’**) (counted nuclei marked by asterisks). Note that in *lrp5* morphants pH3 positive cells are reduced in number. (**C**) Quantification of pH3 cell numbers in the neuroepithelium of rhombomeres 4–8. N = 9/11 (wild-type/*lrp5* morphant). *P < 10^−6^, *t*-test. (**D,E**) 20 ss embryos stained for BrdU incorporation. (D) Wild-type embryo, (E) *lrp5* morphant. Frames demarcate area of cell count (roi) and are shown with higher magnification in (**D’,E’**). Note that in *lrp5* morphants, BrdU labeled cells are reduced in number. (**F**) Quantification of BrdU cell numbers in one unilateral neuroepithelium of rhombomeres 4–8. N = 9/11 (wild-type/*lrp5* morphant). *P = 1.05x10^-6^, *t*-test. (**G-J**) *ccnd1* expression in 20 ss embryos. (G,H) Wild-type embryo, (I,J) *lrp5* morphant. Note that *ccnd1* expression levels are increased in *lrp5* morphants. Anterior is to the left in all images.

The size reduction of migratory CNCC streams suggested that cell numbers were affected by *lrp5* deficiency. Premigratory NCCs are highly proliferative and canonical Wnt signaling controls the NCC cell cycle [12]. It has also been reported that NCC delamination is synchronized with the cell cycle [47]. We therefore assessed the proliferative status of neuroepithelial cells in the region of interest (roi), i.e. in rhombomeres 4 to 8 in *lrp5* morphants at 20 ss. For this, we performed immunohistochemical staining for phosphorylated Histone 3 (pH3), which labels nuclei in M-phase (Fig 6A and 6B). WT control embryos showed an average of 42 pH3-positive nuclei in the roi (n = 11), whereas the number of pH3 positive nuclei in the roi of *lrp5* morphant embryos was reduced to an average of 26 cells (37%; *p* < 10^−6^; n = 11; Fig 6C).

To determine the number of cells in S—phase, we analyzed Bromodeoxyuridine (BrdU) incorporation in *lrp5* morphants compared to wild-type controls at the same developmental stage and in the same roi. It was reported earlier that NCCs synchronize in S-phase before delamination from the neuroepithelium [47], thus being a prerequisite for the start of migration. WT embryos on average displayed 148 BrdU positive cells in the roi (n = 9). In contrast, *lrp5* morphants on average only had 95 BrdU positive cells in the roi (36%; n = 11) indicating a reduction of S-phase nuclei (*p* = 1.05x10^-6^; Fig 6D–6F).

To examine which cell cycle phase is affected in *lrp5* morphants, we analyzed *cyclin d1* (*ccnd1*) transcript levels. *ccnd1* is expressed in G1 and responsible for G1/S-transition. It has been shown to be under positive transcriptional control of Wnt signaling [48], also in zebrafish neural crest [46]. Unexpectedly, *ccnd1* was significantly up-regulated in the hindbrain of 68% (n = 22) of *lrp5* morphants (Fig 6G–6J). This accumulation of *ccnd1* transcripts suggests a possible G1 cell cycle arrest in hindbrain cells of *lrp5* morphants. Consequently, this could lead to the reduced number of cells in S-phase as observed in *lrp5* morphants (Fig 6E).

### Reduced CNCC migratory streams cause cranial skeleton defects

**Fig 7:**
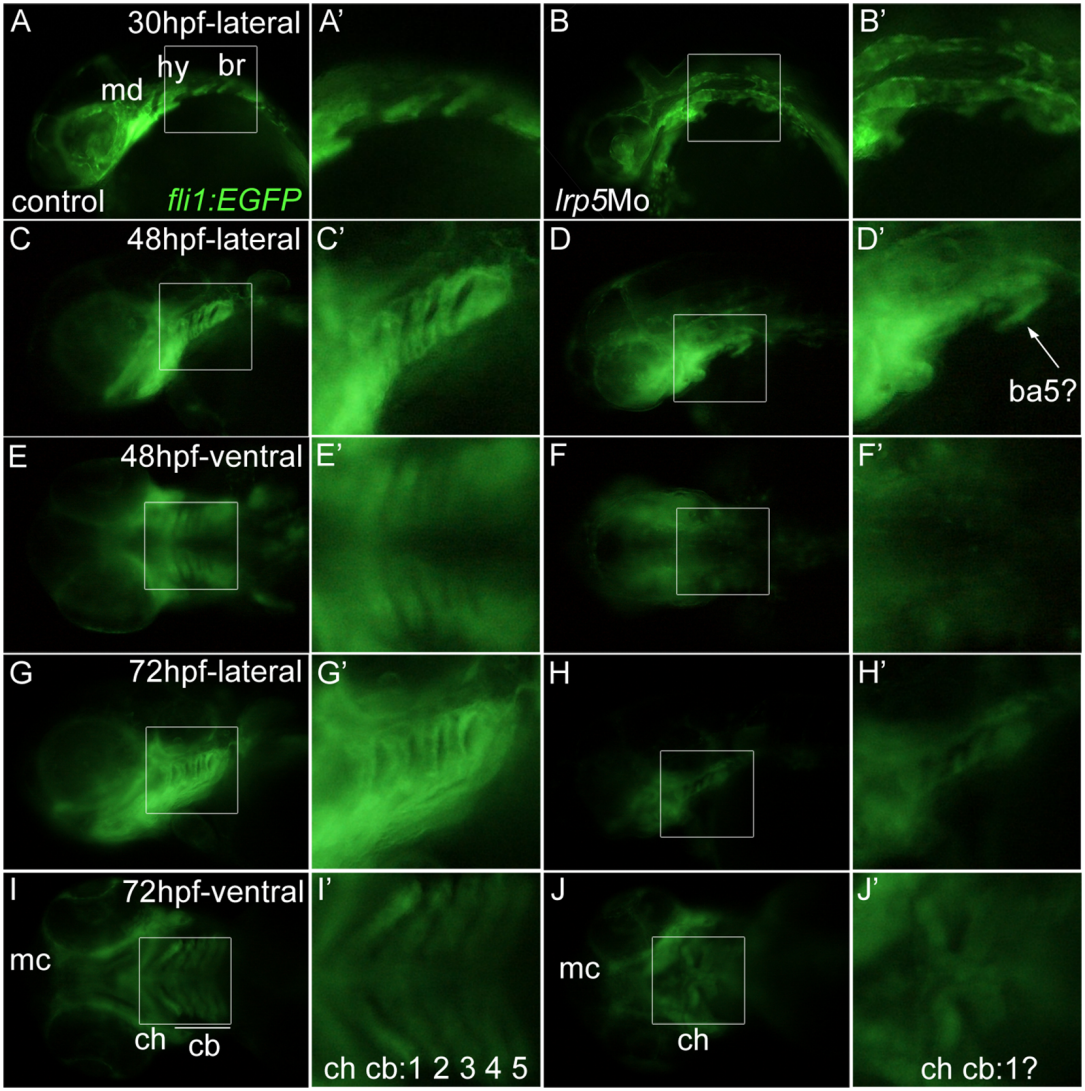
Lower numbers of postmigratory CNCCs after *lrp5* knock-down results in cranial skeleton malformations. (**A-B’**) *fli1*:EGFP embryos at 30 hpf. (A,A’) Uninjected control embryo, (B,B’) *lrp5* morphant. Note that mandibular (md), hyoid (hy) and three branchial (br) patches of postmigratory CNCCs are well defined in wild-type but defective in *lrp5* morphants. (**C-F’**) *fli1*:EGFP embryos at 48 hpf. (C,C’) Uninjected control embryo in lateral view, (D,D’) *lrp5* morphant lateral view, (E,E’) uninjected control embryo ventral view, (F,F’) *lrp5* morphant ventral view. Note that metameric morphology of pharyngeal arches is absent in *lrp5* morphant. Only one arch, most likely the 5^th^ branchial arch is present (ba5?). (**G-J’**) *fli1*:EGFP embryos at 72 hpf. (G,G’) Uninjected control embryo in lateral view, (H,H’) *lrp5* morphant lateral view, (I,I’) uninjected control embryo ventral view (J,J’) *lrp5* morphant ventral view. Note that in wild-type, cranial elements like Meckel’s cartilage (mc), ceratohyal (ch) and 1^st^ to 5^th^ ceratobranchials (cb 1–5) can be distinguished, whereas in *lrp5* morphant only mc and ch are detectable while cbs are undefined. Anterior is to the left in all images. Boxed areas in X are magnified in X’.

Postmigratory CNCCs, once they reach their final destinations, differentiate to establish the head skeleton. We tested whether the observed defects in CNCC proliferation and migration result in reduced numbers of postmigratory CNCCs in the pharyngeal arches. For this, we knocked-down *lrp5* in *fli1*:EGFP transgenic zebrafish that express GFP in CNCC derivatives as well as vascular endothelial cells [27]. At 30 hpf, the mandibular (md), hyoid (hy) and three branchial (br) patches of postmigratory CNCCs show distinct GFP expression in wild-type embryos (Fig 7A). In contrast, 65% of the *lrp5* morphants (n = 32) showed disturbed organization in this area (Fig 7B). We followed development in the affected embryos over time and analyzed morphogenesis and position of GFP positive CNCC derivatives. At 48 hpf, pharyngeal arches were well established in the caudal head region of control embryos and visible as five clearly distinguishable columns of GFP positive cells (Fig 7C and 7E). In contrast, *lrp5* morphants failed to establish proper pharyngeal arch morphology. Only one group of migratory GFP-positive cells could be identified in the posterior hindbrain and most likely represented the 5^th^ branchial arch (ba5; Fig 7D and 7F). At 72 hpf, the majority of CNCC derivatives have reached their final destinations and the distribution of GFP-positive cells reflects the main architecture of the mature ventral cranial skeleton. Structures such as Meckel’s cartilage (mc), ceratohyal (ch) and the five ceratobranchials (cb) are distinguishable (Fig 7G and 7I). In *lrp5* morphants, on the other hand, pharyngeal arches are absent and severe malformations are observed in the cranial skeleton. Only rudiments of the caudal pharyngeal arches remain (Fig 7H). While most parts of the anterior head skeleton are visible in ventral views (mc; Fig 7J), more posterior structures are morphologically not distinguishable (ch, cb; Fig 7J). Together, this suggests that a *lrp5* knock-down initially leads to proliferation defects in premigratory CNCCs, consequently leading to reduced migratory CNCC streams and decreased numbers of postmigratory CNCCs, which ultimately results in morphological defects in the cranial skeleton.

## Discussion

### A role for *lrp5* in morphogenesis of the zebrafish craniofacial skeleton

In this study, we provide the first analysis of expression and activity of the Wnt co-receptor Lrp5 during zebrafish craniofacial development. Sequence alignments showed that zebrafish *lrp5* is highly conserved with significant similarities to *lrp5* sequences in other vertebrates as well as its ortholog *arrow* in *Drosophila*. Elevated and regionally restricted expression of *lrp5* in the early hindbrain are a first hint that *lrp5* might be involved in CNCC formation and migration. As reported previously in zebrafish [46], CNCCs not only derive from an area lateral to the neuroepithelium but also from the neuroepithelium itself, where notably *lrp5* is expressed at this stage. CNCC migration starts at around 14 hpf and results in three distinct streams of migrating cells on both sides of rhombomeres 2, 4 and 6. Thus, *lrp5* expression is found in areas with forming and migrating CNCCs. As development progresses, its expression remains associated with CNCC derivatives as they form the cartilage elements of the ventral cranial skeleton. Interestingly, the overall spatiotemporal expression of *lrp5* in brain and the developing cranial skeleton corresponds well with that of *Sost* [49], a Wnt antagonist known to exert its function by binding to Lrp5 [25]. This suggests that also in teleosts both proteins might interact to control Wnt signaling.

In zebrafish *lrp5* morphants, the most severe defects in viscero-cranial development were observed in ceratobranchials 1–4, while the 5^th^ ceratobranchial containing pharyngeal teeth and other dermal skeletal elements, such as cleitra and operculae, appeared unaffected at least in classI morphants, further underlining the complex role of Wnt signaling in zebrafish pharyngeal tooth formation [50]. In contrast to ceratobranchials 1–4, the 5^th^ ceratobranchials consist mostly of *sox10*:GFP-negative cells (data not shown). Pharyngeal teeth start to form from the pharyngeal epithelium lining the floor of the pharyngeal cavity opposite the ceratobranchials 5 [51] and no *sox10*:GFP positive cells are present in this region. Dentition was normal in *lrp5* morphants and teeth formed in correct positions and with regular alignment according to the developmental stage. The stage of dentition was slightly delayed compared to wild-type, and corresponded to dentition at 56 to 72 hpf. Taken together, the absence of ceratobranchials 1–4 and presence of the 5^th^ ceratobranchial with normal dentition suggested that the effect of the *lrp5* knock-down was restricted to rostral, *sox10* positive subsets of CNCC derivatives in the ventral cranial skeleton.

**Fig 8:**
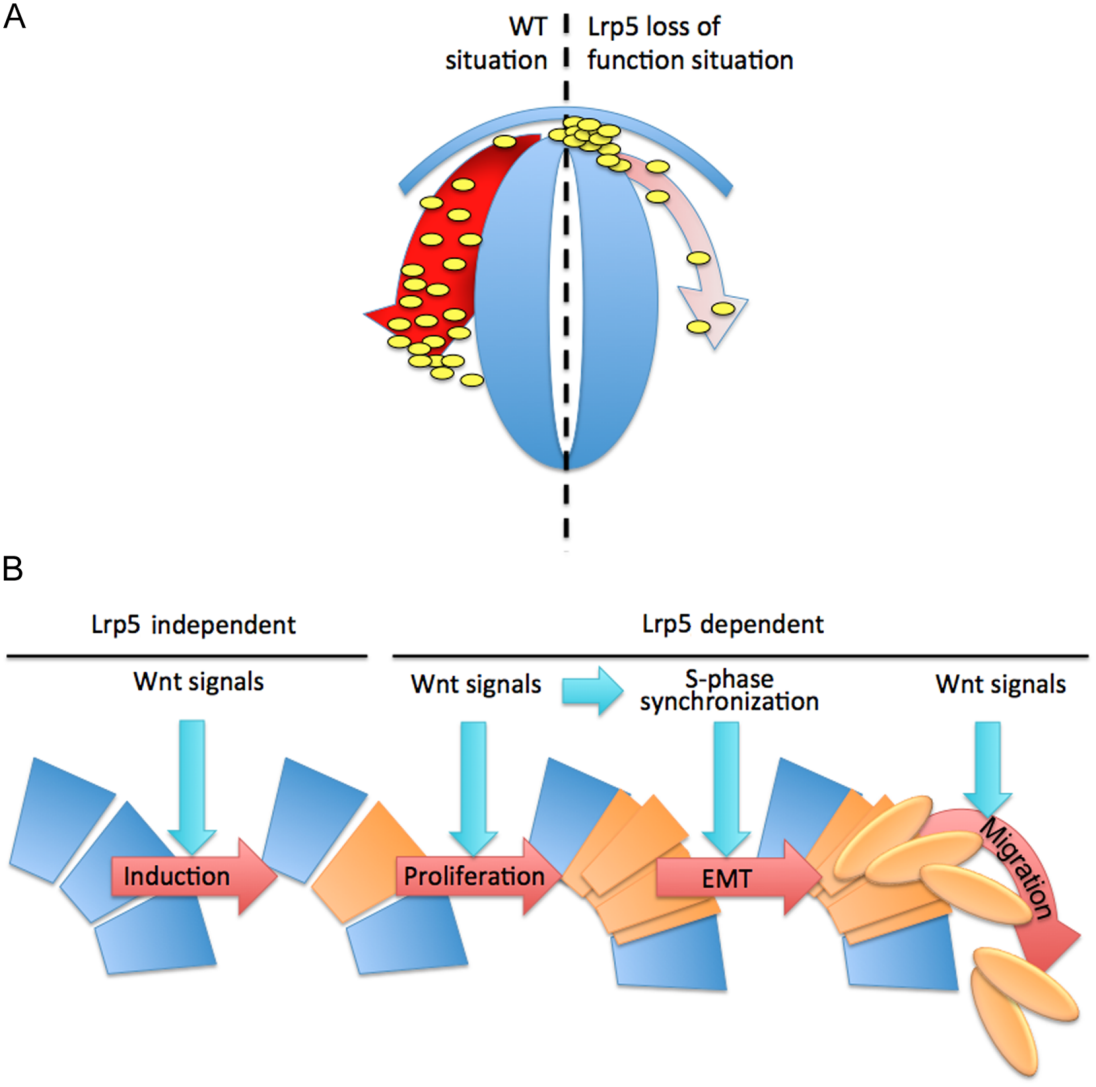
Schematic interpretation of proposed model. (**A**) Comparison between wild-type (WT) and *lrp5* deficient situation. Whereas in wild-type cells migrate, in *lrp5* morphants they are trapped dorsally. (**B**) Wnt signaling is known to be involved in all four steps of NCC development. Induction of CNCCs (orange) seems to occur independent of Lrp5 activity. Proliferation and migration appears to be dependent on Lrp5 mediated Wnt signaling. EMT could indirectly be affected by Lrp5 mediated cell cycle control.

We hypothesize that the induced skeletal defects resulted from events occurring earlier in development. Wnt signaling is involved in different steps of NCC development including NCC induction, also in zebrafish [10]. However, although *lrp5* is expressed at the right place during NCC induction, it seems dispensable for this process, as the pattern of premigratory CNCCs was not affected in morphants. It is possible that our MO data reflect a hypomorphic condition due to incomplete knock-down by the used MOs. However, also after *lrp5* CRISPR/Cas9 injection, normal *foxd3* expression was observed therefore strongly suggesting that *lrp5* is not required for NCC induction. This is particularly interesting since misexpression of a truncated dominant-negative Lrp6 variant in *Xenopus* leads to reduced NCC induction [14]. In *lrp5* morphants, on the other hand, we observed aberrant localization of migratory CNCCs at more advanced stages. At 20 ss, when CNCCs have evaded from the neuroepithelium in wild-type embryos, cells of the branchial stream were retained in the dorsal regions of r6. These cells were identified as NCCs as they expressed *crestin*, *sox10* and *dlx2a* [52]. Importantly, *dlx2a* is only expressed in migratory CNCCs. This therefore suggests that upon *lrp5* knock-down early CNCCs had already completed EMT but failed to initiate migration towards the prospective location of pharyngeal arches. It remains a possibility that the observed ectopic CNCCs are a consequence of a delay rather than a complete stop in migration. In *lrp5* CRISPR/Cas9 injected embryos, we found embryos where *crestin* positive cell clusters spread from medial to lateral positions (see Fig 5K). This could be suggestive for a delay in migration of individual cells. Alternatively, this pattern could also reflect possible defects in collective CNCC migration, where individual mutant cells affect neighboring wild-type cells in a migrating mosaic cell cluster. Based on our observations, we conclude that canonical Wnt signaling through Lrp5 is essential to initiate CNCC migration (Fig 8), as has been proposed from previous experiments *in vitro* [13]. In zebrafish, it had been shown that Wnt signaling affects N-cadherin localization through Ovo1 and thereby regulates NCC migration [53]. Wnts also activate *snail*, which is a repressor of E-cadherin [54]. Thus, the observation that CNCC migration is deficient in zebrafish *lrp5* morphants and after CRISPR/Cas9 injection provides a first hint as to how cell migration is regulated by Wnt signaling in the cranial neural crest.

Delamination of NCCs is tightly intertwined with the cell cycle. Delaminating cells are synchronized in S-phase in a process mediated by Wnts [12]. In *lrp5* morphants, the cell cycle of premigratory CNCCs appeared arrested with reduced numbers of rhombomere cells in M- and S-phase. This suggests a possible involvement of *lrp5* in cell cycle control of premigratory CNCCs. Comparable phenotypes, i.e. reduced numbers of S-phase nuclei in premigratory CNCCs, were reported after expressing a dominant suppressor variant of TCF to inhibit Wnt signaling [46]. However, it remained unclear which Wnt components were responsible for this defect. Wnts regulate the cell cycle through transcriptional regulation of *ccnd1* and thereby control G1/S-phase transition [46,48]. In our studies, we found that *ccnd1* expression was significantly up-regulated in *lrp5* morphants. The elevated *ccnd1* levels in parallel with reduced numbers of cells in S-phase suggest an arrest during G1/S-phase transition. We speculate that there is a feedback on the *ccnd1* promoter in the arrested cells resulting in accumulation of *ccnd1* transcripts. Importantly, a comparable situation has been described in cell cultures where RNAi mediated inhibition of LEF1 also resulted in the up-regulation of Cyclin D1 [55]. Noteworthy, migration defects such as observed in our study are not necessarily a direct consequence of reduced proliferation of premigratory CNCCs and impaired S-phase synchronization. Knock-down of Semaphorin3D in zebrafish for example resulted in reduced proliferation of premigratory CNCCs yet importantly did not lead to a loss of migratory behavior [46]. This suggests that migratory properties of CNCCs are not directly linked to proliferation and that the migration defects observed in this study could also be caused by an independent function of Lrp5 in controlling migratory properties of CNCCs. We therefore speculate that Lrp5 mediated Wnt signaling could be essential for multiple steps in CNCC migration, including proliferation of premigratory CNCCs, S-phase synchronization during EMT as well as migration of delaminated CNCCs (Fig 8). Our finding that zebrafish *lrp5* is involved in proliferation but not specification of CNCCs is consistent with findings for mouse Lrp5, which is required for proliferation of osteoblast precursors but not their differentiation [21].

The morphogenetic fates of individual migratory CNCCs streams have been described earlier [3]. We employed the *fli1*:EGFP transgenic line to follow the fate of postmigratory cells in the craniofacial skeleton of *lrp5* morphants. In contrast to *sox10*:GFP, where GFP expression is restricted to NCCs, the *fli1* line shows EGFP expression in NCCs and the entire pharyngeal arches, thus revealing information about the overall morphology of the cranial skeleton. This allowed us to visualize how the lack of branchial CNCCs resulted in deficient ceratobranchial morphogenesis and revealed that morphogenesis of the 5^th^ ceratobranchial was less affected in *lrp5* morphants.

### A teleost specific function for *lrp5* in craniofacial development?

Present studies on Lrp5 functions are limited to bone metabolism and eye vascularization [17,22]. Loss-of-function mutations in Lrp5 cause the osteoporosis pseudoglioma (OPPG) syndrome [21]. Surprisingly, no craniofacial deficiencies or any other neural crest related abnormalities were reported in Lrp5 knock-out mice. However, observations in human patients suffering from genetically inherited LRP5 gain-of-function mutations reported mild aberrations in skull anatomy [25]. Some of these patients are characterized by abnormally thickened jaws or lobulated palates from earliest ages onwards. Patients with a specific gain-of-function mutation (A214T) also suffer from craniosynostosis [24]. The early onset of these deformations suggests that they are not a result of a progressive sclerosteosis as described in other Lrp5 gain-of-function mutants. As jaw, palate and skull originate from the CNCC, it is thus tempting to speculate that the symptoms observed in human patients are caused also by neural crest aberrations that occurred during embryogenesis.

Our observations suggest a role for Lrp5 in craniofacial morphogenesis that appears more imperative in zebrafish than compared to its role in mammals. Lrp5 knock-down in fish resulted in severe craniofacial defects compared to the milder defects described in mouse models or human patients. We found that the most severely affected structures in the craniofacial skeleton were the ceratobranchials that support the gills. These structures derive from branchial streams of CNCCs that shifted their morphogenetic destination in the course of vertebrate evolution. In amphibians, they generate cells that eventually build up bones of the skull proper [56]. During human embryonic development, however, branchial NCCs play a less important role in cranium formation and contribute to the formation of squamosal, alisphenoid and hyoid bones [57]. It is therefore tempting to speculate that the different destinations of migratory CNCCs during branchial morphogenesis in various vertebrates reflects a modification or possible neo-functionalization of *lrp5*’s role in teleosts and other non-mammalian vertebrates.

## Supporting Information

S1 Fig (**A**)Schematic representation of *lrp5* transcript and Mo knock-down strategy. (**B**) Semi-quantitative RT-PCR in morphant embryos, +/- indicates presence or absence of reverse transcriptase. (**C**) Table presenting distribution of phenotypes upon injection with *lrp5* MOs. (**D**) Graphical presentation of data shown in (C). (**E-L**) 20 ss embryos stained for different transcripts. (E,F) Wild-type embryo stained for *dlx2a*. (G,H) *lrp5* mismatch morphant stained for *dlx2a*. (I,J) Wild-type embryo stained for *crestin*. (K,L) *lrp5* mismatch morphant embryo stained for *crestin*. Note that mismatch Mo injection does not result in alterations of *dlx2a/crestin* expression patterns. Anterior is to the left in E-L.
(TIF)

S2 Fig *lrp5* CRISPR2 injected embryos display normal induction of CNCCs, but show defects in the craniofacial skeleton. (A-D) *foxd3* expression at 10 ss in wild-type embryos (A,B) and *lrp5* CRISPR2 injected embryos (n = 20/20 with normal *foxd3* expression; C, D). (A,C) are lateral views with anterior to the left, (B,D) are dorsal views. (E-H) Additional examples for embryos with varying degrees of cartilage defects (class I, F; class II, G; class III, H). Combined bone and cartilage staining at 7 dpf of wild-type (E) and *lrp5* CRISPR1+2 injected embryos.
(TIF)
