# Supplementary figures and images for: The Wnt Co-Receptor Lrp5 Is Required for Cranial Neural Crest Cell Migration in Zebrafish
Source: PLoS One. 2015 Jun 29;10(6):e0131768. doi: 10.1371/journal.pone.0131768 (PMC4486457; doi:10.1371/journal.pone.0131768)

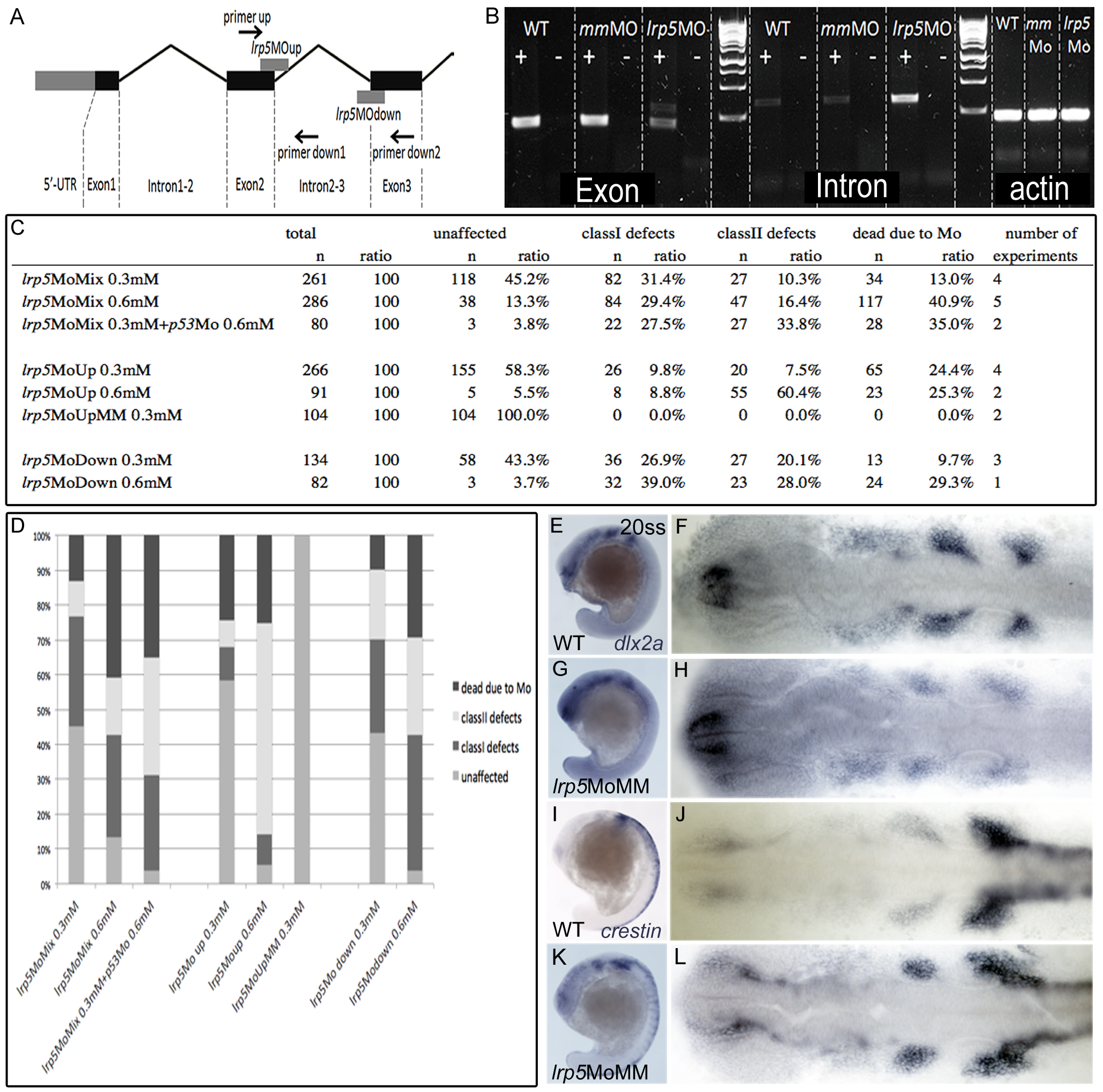

Supplement: S1 Fig — (A)Schematic representation of lrp5 transcript and Mo knock-down strategy. (B) Semi-quantitative RT-PCR in morphant embryos, +/- indicates presence or absence of reverse transcriptase. (C) Table presenting distribution of phenotypes upon injection with lrp5 MOs. (D) Graphical presentation of data shown in (C). (E-L) 20 ss embryos stained for different transcripts. (E,F) Wild-type embryo stained for dlx2a. (G,H) lrp5 mismatch morphant stained for dlx2a. (I,J) Wild-type embryo stained for crestin. (K,L) lrp5 mismatch morphant embryo stained for crestin. Note that mismatch Mo injection does not result in alterations of dlx2a/crestin expression patterns. Anterior is to the left in E-L. (TIF) [file pone.0131768.s001.tif]

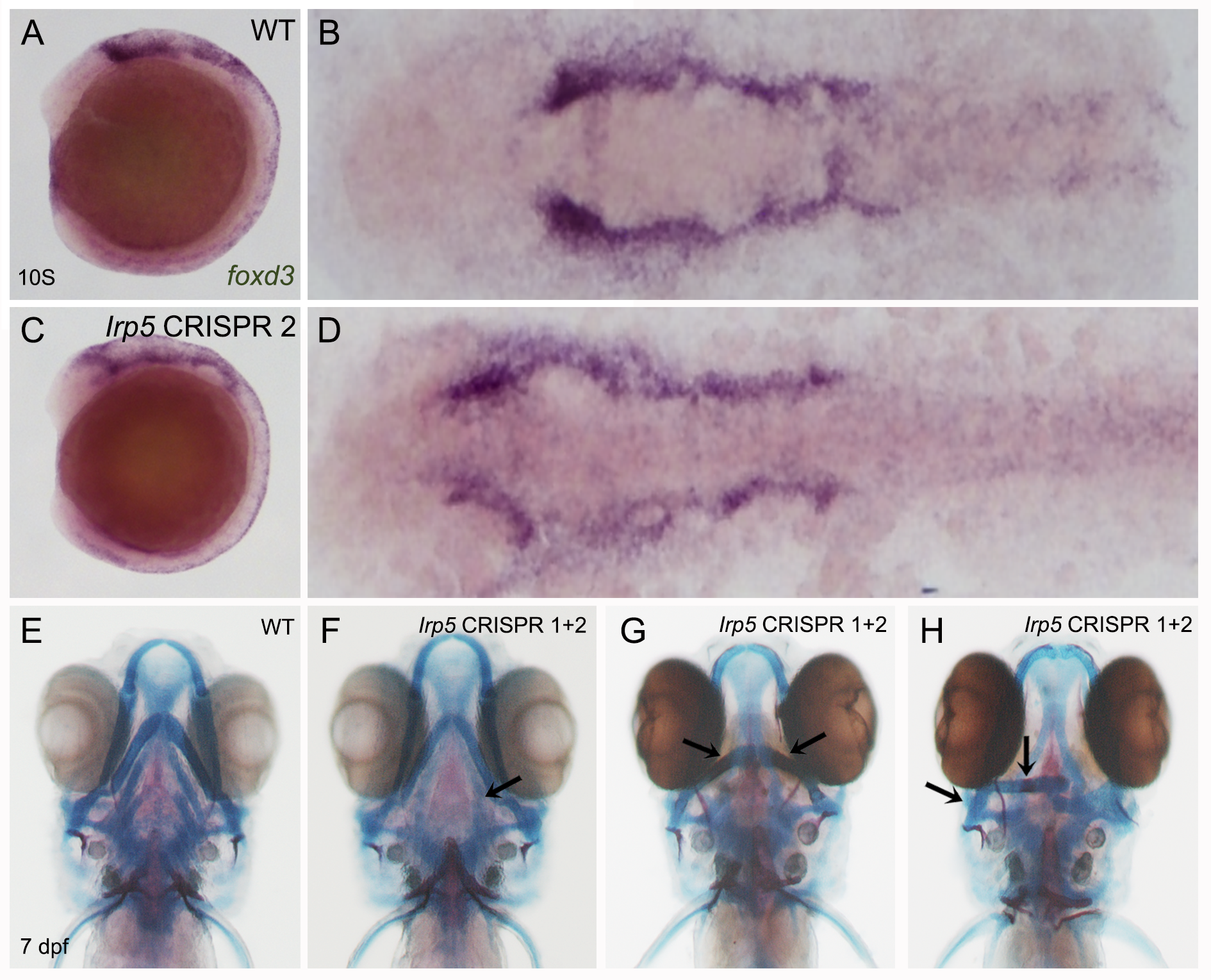

Supplement: S2 Fig — (A-D) foxd3 expression at 10 ss in wild-type embryos (A,B) and lrp5 CRISPR2 injected embryos (n = 20/20 with normal foxd3 expression; C, D). (A,C) are lateral views with anterior to the left, (B,D) are dorsal views. (E-H) Additional examples for embryos with varying degrees of cartilage defects (class I, F; class II, G; class III, H). Combined bone and cartilage staining at 7 dpf of wild-type (E) and lrp5 CRISPR1+2 injected embryos. (TIF) [file pone.0131768.s002.tif]
